# Diagnostic performance of point-of-care and central laboratory cardiac troponin assays in an emergency department

**DOI:** 10.1371/journal.pone.0188706

**Published:** 2017-11-28

**Authors:** Petra Wilke, Annette Masuch, Oliver Fahron, Stephanie Zylla, Tobias Leipold, Astrid Petersmann

**Affiliations:** 1 Central Emergency Department, Klinikum Frankfurt (Oder) GmbH, Rhön AG, Frankfurt (Oder), Germany; 2 Institute of Clinical Chemistry and Laboratory Medicine, University Medicine Greifswald, Greifswald, Germany; 3 ClinPath GmbH, Berlin, Germany; The University of Tokyo, JAPAN

## Abstract

Early diagnosis of myocardial infarction (MI) with cardiac troponin (cTn) assays at the point-of-care (POC) is suggested to shorten turn-around-time in the emergency department (ED). The present study aimed at comparing the diagnostic performance of two POC cTn assays with that of a central laboratory high-sensitivity (hs) method, under routine ED conditions. In 2,163 non-selected ED patients suspected for MI, the diagnostic performance of the POC troponin I (TnI), troponin T (TnT), and hs-TnT assay for the prediction of MI was evaluated based on receiver operating characteristic (ROC) analyses and compared with the performance based on the manufacturers’ cut-offs. Due to an observed association between renal function as determined by estimated glomerular filtration rate (eGFR) and cTn concentrations, all analyses were stratified by renal function. In patients with normal renal function (eGFR > 60 mL/min/1.73m^2^), POC and hs assays showed a comparable diagnostic performance as quantified by the area under the ROC curve (AUC) of about 0.88. The ROC-derived optimal cut-off (OCO) levels for the different cTn assays clearly changed with decreasing kidney function. Impaired kidney function required OCO to be three to five times higher to achieve a comparable performance. Particularly cTnT concentrations were strongly associated with renal function. The three cTn assays demonstrated equivalent diagnostic performance in ED-patients admitted with suspected ACS in relation to the release diagnosis, supporting the use of POC testing in this setting. The present results implicate that application of eGFR-specific OCOs may decrease false-positives among patients with impaired renal function. Providing individual cut-offs depending on patients’ eGFR might be an appropriate add-on tool to improve specificity in the diagnosis of MI.

## Introduction

Acute coronary syndrome (ACS) is a common cause for patients attending the emergency department (ED). The early diagnosis of myocardial infarction (MI) and appropriate immediate treatment is important to minimize myocardial injury. Currently, the use of cardiac troponin (cTn) is the reference standard in MI diagnostics [1]. The 99^th^ percentile of cTn determined in a presumably healthy reference population plays a central role as the cut-off for the diagnosis. According to the third universal definition of myocardial infarction, the hallmark of myocardial necrosis is determined by the rise and/or fall of at least one value above the 99^th^ percentile of cTn in serial measurements [1]. Detection of cTn concentrations below the 99^th^ percentile in more than 50% of a healthy reference population is the definition of an assay as high sensitive (hs) [2]. In addition, cTn assays are evaluated with respect to their analytical performance, i.e. imprecision at the 99^th^ percentile which preferably should be ≤ 10% [1,2]. cTn assays in point-of-care (POC) settings often do not perform as well as those available in a central laboratory and would not be accepted as hs-cTn. Consequently, POC cTn assays may have a limited first-draw diagnostic sensitivity due to the time required for a detectable rise in measurable cTn concentrations [3].

cTn measurements are recommended at admission and should be repeated 3–6 hours later [1]. As a result, the majority of ED patients require prolonged assessment prior to safe discharge [4]. This may be associated with significant healthcare costs and ED overcrowding [5].

It is a major challenge to correctly identify patients who should be monitored for suspected MI. There are complaints typical for ACS like pressure-type chest-pain which may be accompanied by sweating, nausea, abdominal pain, dyspnea, and syncope, all indications for assessment by cTn measurements [3]. Atypical symptoms include epigastric pain, indigestion-like symptoms, and isolated dyspnea [3] that may indicate a silent MI [6]. The highest incidence, prevalence, and adverse outcomes of ACS are found in elderly patients who also have atypical symptoms more frequently. Thus, it is recommended to consider non-ST-segment elevation MI (NSTEMI) in this patient group already at slightest suspicion [3].

The present study evaluated the potential of POC assays for early MI diagnosis in an unselected population of ED patients. We compared the diagnostic performance of two contemporary sensitive POC cTn assays (POC TnI and POC TnT) with a hs-TnT assay for the prediction of MI. Because research provides evidence for a relation between cTn and kidney function [7–9], influence of renal function on the diagnostic performance was considered. The data from this ED population were used to evaluate the relation between complaints at admission, the age of the patients and the prevalence of MI. This retrospective study confirms previous reports of increased cTn with a decreased kidney function. This leads to higher numbers of false positive results i.e. a decreased specificity of the assay. Receiver operating characteristic (ROC) analyses were used to derive optimized cut-off values to improve specificity of cTn assays. The use of individual cut-off values considering kidney function also increased the positive likelihood ratio.

## Materials and methods

### Study design and patients

During 2012, 25,511 patients were admitted to the ED of the general district hospital in Frankfurt (Oder), Germany of which 3,743 (14.7%) presented primary symptoms indicative for ACS. Classification of patients was guided by the triage software ERPath (ClinPath, Berlin) based on the Manchester Triage System and was documented in the hospital information system (iMedOne telekom Healthcare Solutions). This triage system assigns a default symptom diagram (e.g. chest pain, dyspnea, palpitation, syncope and non-specific complaints like dizziness, general discomfort, etc.) to the patient. If a patient was classified into a category requiring troponin measurements according to the triage system, POC cTnI was determined from whole blood samples within 15 min. POC cTnT was determined in the same blood sample for the purpose of this study only. A parallel sample from the same venous puncture was sent to the central laboratory for further analysis including hs-TnT concentration. For immediate diagnostic purposes in the ED only the POC cTnI results were used whereas the results of the hs-TnT were requested by the cardiology department where further treatment took place. The primary endpoint for this restrospective study was the release diagnosis, MI or non-MI. The final diagnosis of MI was decided by a cardiologist considering all available diagnostic information about the patient. Patients were not followed-up.

Only patients with available data on sex, age, cTn for all three assays, creatinine serum concentrations, and information on the final diagnosis were included in the analysis (n = 2,163). A subsample from this population was selected without cardiac or renal impairment:, i.e. Nt-proBNP concentration < 125 ng/L, and eGFR ≥ 60 mL/(min×1.73 m^2^) (n = 573). Elevated levels of the N-terminal fragment cleaved from the prohormone of B-type natriuretic peptide (Nt-proBNP) was chosen as a criterion to exclude ED patients at potential risk for myocardial dysfunction because of its role in heart failure rule-out, its proven good prognostic value in several cardiac diseases[10], and its pronounced effect on determination of the 99^th^ percentile of cTnI in a presumably healthy population[11].

The Ethics committee of the federal state Brandenburg was informed on the study by submission of the study protocol. The Ethics committee informed us, that in accordance with the German law, no individual approval of the study was needed. Moreover, the committee members decided, that no written consent of the patients was necessary for the measurement of a second cTn type in the whole blood sample taken in the ED for diagnostic purposes. In 2012, when the study was conducted, the German law did not request additional written consent from patients for the analysis of anonymized patient care data. The study is a retrospective evaluation of the data of the year 2012.

### Methods and measurements

Measurements of the POC troponin cTnI and cTnT concentrations were made on the AQT90 FLEX platform (Radiometer, Willich, Germany) using whole blood samples. The manufacturer’s recommended cut-off concentrations for the AQT90 FLEX cTnT and cTnI assays were 17 ng/L and 23 ng/L, respectively (%CV < 20%). The hs-TnT assay was performed in the central laboratory on Cobas 6000 (Roche Diagnostics, Mannheim, Germany) applying the Elecsys Troponin T high sensitive assay. The manufacturer assigned the 99^th^ percentile as 14 ng/L (%CV < 10%). Serum creatinine concentrations were determined also using COBAS 6000 and the Jaffe reaction. The glomerular filtration rate (GFR) was estimated by the Chronic Kidney Disease Epidemiology Collaboration (CKD-EPI) equation[12].

In an ED setting, GFR needs to be estimated from a creatinine concentration. The CKD-EPI equation can only quantify values below 60 mL/min/1.73m^2^ [12]. Therefore, normal renal function was assumed if the eGFR was ≥ 60 mL/min/1.73 m^2^, impaired renal function was assumed for an eGFR ≥ 30 and < 60 mL/min/1.73m^2^, and an eGFR < 30 mL/min/1.73m^2^ was considered as severely restricted renal function. Plasma concentrations of Nt-proBNP were measured using the Nt-proBNP assay on the AQT90 FLEX analyzer. For all assays of the study, quality control was performed according to the Guideline of the German Medical Association on Quality Assurance in Medical Laboratory Examinations (Rili-BAEK) [13,14].

### Statistical analyses

Data are expressed as median (25^th^; 75^th^ quartile), and sex distribution is given as percentage. Chi^2^-test (sex distribution) or Mann-Whitney U test (non-parametric data) was used to compare patients with MI and those without MI. Because patients aged > 75 years were reported to present more often atypical complaints bearing the risk of miss-diagnosis[15,16], the subjects complaints were listed separately in patients aged ≤ 75 years and those who were older. To evaluate the diagnostic performance of the three different cTn assays for the prediction of MI, ROC analyses were used. Logistic regression models with MI as outcome and the cTn assay as predictor were estimated and the interaction between the respective cTn and eGFR was tested. As all models revealed significant interaction terms, the analyses were stratified by three eGFR groups (normal [n = 1,376], impaired [n = 603], severely restricted [n = 184] renal function). The sensitivity of each cTn test over all possible false-positive rates was displayed in ROC curves and the area under the curve (AUC) was estimated as a measure of discrimination. To obtain the optimized cut-off (OCO) value, the distance (d) between the point (0, 1) and any point on the ROC curve was minimized: ($Min(d)=\sqrt{{\left(1-sensitivity\right)}^{2}+{\left(1-specificity\right)}^{2}}$). 95%-confidence intervals were determined using bootstrap analysis. The analytical performance of the cTn assays in each of the three eGFR groups was characterized by the sensitivity, specificity, false positive rate (FPR, 1-specificity), false negative rate (FNR, 1-sensitivity), positive predictive value (PPV), negative predictive value (NPV), and positive likelihood ratio (LR+).

For comparisons, the diagnostic performance of the cTn assays in the three different eGFR groups was also determined by using the manufacturers’ recommended cut-off values. Simple binomial proportion method (Clopper-Pearson estimation method) was applied to compute a 95%-confidence interval for sensitivity, specificity, FPR, FNR, PPV, and NPV.

The subsample of patients without renal and cardiac impairment was used to determine the 99^th^ percentile of each cTn distribution.

A p-value < 0.05 was considered statistically significant. Statistical analyses were performed using SAS 9.4 (SAS Institute Inc., North Carolina, USA).

## Results

### General characteristics of the study population

In our study population 125 of 2,163 patients (5.8%) had MI. Patients with MI were more often men and had significantly higher creatinine and cTn values (Table 1). The median age was 71 years and 797 patients (37%) were above 75 years of age. Table 2 presents an overview of the subjects’ complaints when admitted to the ED according to age and MI. Chest pain is a typical complaint for MI and the majority of MI diagnosis was adjudicated in patients that were admitted with this complaint. This was observed less often in the elderly (56.8%) than in the younger subjects (85.2%). Dyspnea was the second leading complaint in patients with MI in younger as well as elder patients. Notably, a plurality of all patients (27.9%) was admitted with unspecific complaints. The proportion of MI diagnoses in the younger was rather low (1.2%). Elderly patients more often indicated unspecific complaints when they had MI (13.6%, Table 2).

**Table 1:** Descriptive statistics of the study population.

	without MI	MI	p-value
	n = 2038	n = 125	
age	71 (57; 79)	70 (59; 80)	0.83
sex (% men)	51.9	63.2	0.01
laboratory hs-TnT (ng/L)	10 (9; 23)	84 (37; 252)	< 0.01
POC TnT (ng/L)	9 (9; 16)	59 (24; 270)	< 0.01
POC TnI (ng/L)	9 (9; 9)	74 (21; 340)	< 0.01
creatinine (mg/L)	0.97 (0.81; 1.22)	1.04 (0.87; 1.28)	0.04
eGFR (ml/(min*1.73m^2^))	70.9 (49.6; 88.7)	66.2 (48.9; 86.3)	0.26

Data are expressed as median (25^th^; 75^th^ quartile); sex distribution is given as percentages. Chi^2^-test (nominal data) or Mann-Whitney U test (continuous data) was used for comparisons between patients without myocardial infarction (MI) diagnosis and those with MI diagnosis. eGFR was calculated using CKD-EPI_creat_ equation. eGFR = estimated glomerular filtration rate; hs = high sensitivity; TnT = troponin T; TnI = troponin I; POC = point-of-care; creat = creatinine.

**Table 2:** Subjects’ complaints when admitted to the emergency department.

	age ≤ 75			age > 75		
	without MI	MI	p-value	without MI	MI	p-value
	n = 1285	n = 81		n = 753	n = 44	
Complaints (%)			< 0.01			< 0.01
abdominal pain	11.9	0		6.9	4.6	
chest pain	23.8	85.2		15.4	56.8	
collapse	5.6	3.7		6.7	0	
dyspnea	12.8	9.9		19.1	20.5	
falling	1.7	0		5.7	4.5	
other	17.3	0		12.9	0	
unspecific	26.9	1.2		33.3	13.6	

The patients were allocated to two groups. Data are given as percentages. Chi^2^-test was used for evaluation of the comparisons of patients without and with the diagnosis myocardial infarction (MI).

### Diagnostic performance of the three troponin assays

**Fig 1:**
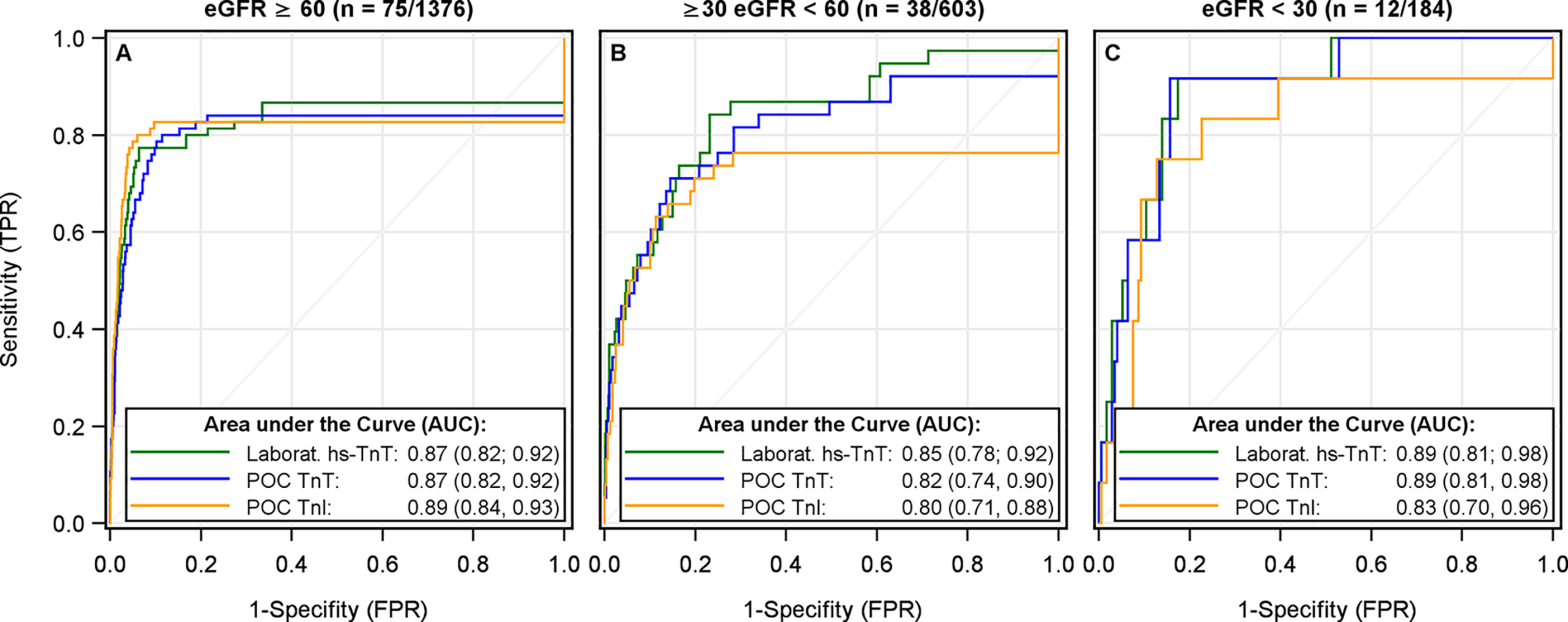
Diagnostic performance of all cTn assays was revealed by receiver operating characteristic (ROC) analyses. Analyses were done separately in renal healthy patients with an estimated glomerular filtration rate (eGFR) ≥ 60 mL/min/1.73m^2^ (A), in patients with an impaired renal function (B), and in patients with severely restricted renal function (C). For each patient group the number of people diagnosed with myocardial infarction in relation to the number of overall patients is given above the respective figure. ROC curves and the resulting area under the curve (AUC) together with its 95% confidence interval are presented for the central laboratory hs-TnT (green), POC TnT (blue), and POC TnI (orange). TPR = true positive rate; FPR = false positive rate.

In patients with normal renal function the AUC derived from ROC analyses showed comparable performance of the cTn assays with 0.87, 0.87, and 0.89 for the hs-TnT, the POC TnT, and the POC TnI assay, respectively (Fig 1A). In patients with impaired renal function a minor drop in the AUC was seen for all cTn assays (Fig 1B). Furthermore, the confidence intervals for the AUCs is large due to the small number of MI cases in this group. In patients with severely restricted renal function the AUC of the results of the hs-TnT and POC TnT assays were slightly increased compared to those found in renal healthy patients. However, the number of MI cases was very small and thus, the confidence intervals large (Fig 1C).

The diagnostic performance of cTn assays using the OCO as determined by ROC analyses is summarized in Table 3, whereas the diagnostic performance of all assays using the manufacturers recommended cut-off values is given in Table 4. Both tables include data on sensitivity, specificity, FPR, FNR, and LR+. S1 and S2 Tables provide PPV, NPV, numbers of true positives, true negatives, false positives, and false negatives as would be recognized in the patient population with either the ROC-derived OCO or the cut-off recommended by the manufacturer, respectively.

**Table 3:** Diagnostic performance of cTn assays using the optimized cut-off (OCO) value as determined by receiver operating characteristic (ROC) analyses.

	OCO	Sens (%)	Spec (%)	FPR (%)	FNR (%)	LR+
**eGFR ≥ 60**	**n = 75/1376**					
Laboratory hsTnT (ng/L)	33 (18; 36)	77 (70; 87)	94 (83; 95)	6 (5; 17)	23 (13; 30)	12.1 (4.5; 16.3)
POC TnT (ng/L)	18 (13; 22)	80 (73; 88)	89 (81; 92)	11 (8; 19)	20 (12; 27)	7.0 (4.2; 10.0)
POC TnI (ng/L)	14 (14; 27)	83 (74; 90)	90 (89; 96)	10 (4; 11)	17 (10; 26)	8.5 (7.4; 20.5)
**30 ≤ eGFR < 60**	**n = 38/603**					
Laboratory hsTnT (ng/L)	33 (29; 43)	84 72; 94)	77 (72; 86)	23 (14; 28)	16 (6; 28)	3.6 (2.9; 6.0)
POC TnT (ng/L)	33 (21; 38)	71 (65; 88)	86 (69; 89)	15 (11; 31)	29 (12; 35)	4.9 (2.4; 7.1)
POC TnI (ng/L)	15 (11; 24)	71 (59; 86)	80 (71; 90)	20 (10; 29)	29 (14; 41)	3.6 (2.4; 7.6)
**eGFR < 30**	**n = 12/184**					
Laboratory hsTnT (ng/L)	100 (100; 202)	92 (73; 100)	83 (78; 93)	17 (7; 22)	8 (0; 27)	5.3 (3.8; 12.9)
POC TnT (ng/L)	97 (97; 190)	92 (73; 100)	84 (79; 92)	16 (8; 21)	8 (0; 27)	5.8 (4.1; 11.3)
POC TnI (ng/L)	46 (13; 180)	75 (57; 100)	87 (0; 93)	13 (7; 100)	25 (0; 43)	5.9 (1.0; 12.6)

Patients were stratified in three groups according their renal function. For each patient group the number of people diagnosed with MI in relation to the number of overall patients is given. The optimized cut-off value (OCO) was determined by ROC analyses. Sensitivity (Sens), Specificity (Spec), false-positive rate (FPR), false-negative rate (FNR), and the positive likelihood ratio (LR+) are shown. In brackets the 95% confidence interval for each parameter is given. Confidence intervals were assessed by using bootstrapping methods. eGFR = estimated glomerular filtration.

**Table 4:** Diagnostic performance of cTn assays using the manufacturers recommended cut-off values (CO mf).

	CO mf	Sens (%)	Spec (%)	FPR (%)	FNR (%)	LR+
**eGFR ≥ 60**	**n = 75/1376**					
Laboratory hsTnT (ng/L)	14	81 (71; 89)	77 (74; 79)	23 (21; 26)	19 (11; 29)	3.5
POC TnT (ng/L)	17	80 (69; 88)	88 (86; 89)	12 (11; 14)	20 (12; 31)	6.5
POC TnI (ng/L)	23	79 (68; 87)	95 (93; 96)	5 (4; 7)	21 (13; 32)	15.1
**30 ≤ eGFR < 60**	**n = 38/603**					
Laboratory hsTnT (ng/L)	14	95 (82; 99)	39 (35; 43)	61 (57; 65)	5 (1; 18)	1.6
POC TnT (ng/L)	17	84 (69; 94)	64 (60; 68)	36 (32; 40)	16 (6; 31)	2.3
POC TnI (ng/L)	23	63 (50; 78)	89 (86; 91)	11 (9; 14)	37 (22; 50)	5.6
**eGFR < 30**	**n = 12/184**					
Laboratory hsTnT (ng/L)	14	100 (74; 100)	6 (3; 10)	94 (90; 97)	0 (0; 26)	1.1
POC TnT (ng/L)	17	100 (74; 100)	22 (16; 28)	78 (72; 84)	0 (0; 26)	1.3
POC TnI (ng/L)	23	83 (52; 98)	76 (69; 82)	24 (18; 31)	17 (2; 48)	3.5

Patients were stratified in three groups according their renal function. For each patient group the number of people diagnosed with MI in relation to the number of overall patients is given. The cut-off value belongs to the manufacturers’ information of the respective cTn assay (CO mf). Sensitivity (Sens), Specificity (Spec), false-positive rate (FPR), false-negative rate (FNR), and the positive likelihood ratio (LR+) are shown. In brackets the 95% confidence interval for each parameter is given. Confidence intervals were assessed by using simple binomial proportion method (Clopper-Pearson estimation method). eGFR = estimated glomerular filtration.

Based on ROC curves the OCO value for each cTn assay in each patient group was determined. In the group of patients with normal renal function the OCO for the hs-TnT assay was considerably higher compared to the cut-off recommended by the manufacturer (33 ng/L vs. 14 ng/L, respectively) and remained at 33 ng/L for patients with impaired renal function. It was up to 100 ng/L in patients with severely restricted renal function.

The OCO of the POC TnT assay in the group of patients with normal kidney function agreed with that recommended by the manufacturer (18 ng/L vs. 17 ng/L, respectively). However, for patients with impaired and severely restricted renal function the OCO was 33 ng/L and 97 ng/L, respectively.

The OCO assessed for POC TnI was considerably lower than the manufacturer’s recommendation (14 ng/L vs. 23 ng/L). In patients with impaired renal function a similar cut-off value for POC TnI was found as in patients with normal renal function (15 ng/L), whereas in patients with severely restricted renal function the ROC-derived OCO increased to 46 ng/L.

After applying the manufacturers’ cut-offs, there was a considerably higher number of false positive measurements for all assays in all groups of renal function. Of note, with the hs-TnT assay even in renal healthy patients the FPR was 23%. This proportion increased in patients with impaired renal function to 61%, whereas in patients with severely restricted renal function the FPR was 94%. Similar to the hs-TnT assay, the FPR increased for the POC TnT assay from 12% in patients with normal renal function to 78% in patients with severely restricted renal function.

By optimizing the cut-offs (Table 3) the loss of specificity in patients with low eGFR as observed with the recommended cut-offs (Table 4) was overcome. Improved diagnostic performance is also illustrated by the LR+ which increased for all three cTn assays when ROC-derived OCOs were applied compared to the results assessed by applying the manufacturers’ cut-off.

### The 99^th^ percentile of cTn assays in a cardiac and renal healthy patient-based population

In view of these data, it was of interest to calculate the 99^th^ percentile in a patient-based population without myocardial and renal impairment (n = 573). The 99^th^ percentile in this patient population was 34 ng/L for the hs-TnT assay, 26 ng/L for the POC TnT assay, and 23 ng/L for the POC TnI assay.

## Discussion

The present study compared the diagnostic performance of two POC cTn assays with one core-laboratory hs-cTn assay in an unselected ED-patient population. In addition, we used data from this population to evaluate the relation between complaints at admission, the age of the patients and the prevalence of MI.

It has been described that renal impairment is associated with elevated troponin plasma concentrations[7–9,17]. It is known that cTnT is eliminated, at least in part, via the kidney whereas cTnI is not[18], but results from various other studies are conflicting. Until to today the etiology of elevated cTn in patients with renal dysfunction is not completely understood[9]. In our study, in renal healthy patients all cTn assays showed a comparable diagnostic performance with an AUC of about 0.88, which is somewhat lower compared to previous reports on diagnostic performance of the hs-TnT assay[17,19] The estimated AUC for the POC TnT and TnI assays was comparable to other reports[20,21]. In line with a previous study[17], the AUCs changed only slightly with decreasing renal function.

Application of the ROC-derived OCOs increased specificity of cTnT assays in all groups of renal function. This reduced FPR considerably while sensitivity was kept at a high percentage resulting in a better diagnostic performance as also demonstrated by higher LR+. By decreasing the number of false positive cTn values more reliable results for a safe rule-out would be provided for patients with renal dysfunction. When applying the cut-offs as recommended by the manufacturers specificity of the cTnT assays was strongly affected with decreasing eGFR. In daily practice, most practitioners would prefer to sacrifice specificity to maintain sensitivity. However, the high FPR translates also into > 75% of patients with severe renal dysfunction but without MI who would be subjected to further ACS diagnostics. The rise and/or fall of cTn in serial measurements has become a major component in the definition of MI because impaired renal function was found to be associated with chronically increased cTn values with low variation in absence of cardiac damage [1]. Nevertheless, positive cTn results in the first assessment lead to serial cTn measurements necessary for delta-detection to rule-in or rule-out MI. Apart from a delayed diagnosis contributing to increasing costs and overcrowding of the ED, the high FPR might have severe consequences for patients without MI. This might be overcome with implementation of optimized cut-off values for patients with kidney dysfunction. Such a concept has been suggested before. Twerenbold et al. determined the OCO for the hs-TnT assay to be 2.1-fold higher than the 99^th^ percentile, which is 29.4 ng/L, being comparable to the ROC-derived OCO assessed in the present study for impaired renal function [17]. The study by Huang et al. examined hs-TnT in hemodialysis patients. In line with our findings they determined the OCO for diagnosis of MI in hemodialysis patients at 107 ng/L at admission to the ED[22]. Of note, even in the patient group with normal eGFR the diagnostic performance of the hs-TnT assay increased considerably when applying the higher OCO. The ROC-derived OCO for the POC TnT assay was very close to the cut-off recommended by the manufacturer in favor of the POC assay in patients with normal eGFR.

Partitioning of cut-off values depending on the age and renal status of the patient seems justified. In the field of laboratory medicine algorithm quotients are relatively common (e.g. the eGFR) and well accepted by physicians. In the ED setting, determination of creatinine simultaneously with cTn at a comparably low cost would mean a time benefit for the patient as it allows providing cTn values combined with an optimized cut-off adjusted according to the patient’s eGFR, which might positively affect quality of patient care. In line with this, Lippi and Cervellin suggested that accurate interpretation of test results would necessitate the development and validation of more specific cut-offs leading ideally to personalized diagnostic thresholds[23].

Notably, cut-offs provided by manufacturers are typically determined as 99^th^ percentiles in a presumably healthy population. However, reference values shall be determined in a population as close as possible to the population in which the test will be applied [24]. Thus, the present data were used to calculate the 99^th^ percentile as observed in a patient-based population without myocardial and renal impairment. Numerous comorbidities are described to increase cTn including hypertension and type-2 diabetes[25] which are very common in patients in the ED. The 99^th^ percentile for the cTnT assays in the patient population was considerably higher compared to the cut-off recommended by the manufacturers implying that in the ED setting a higher cut-off for cTnT assays may be more appropriate. In contrast to the cTnT assays, the 99^th^ percentile for the POC TnI assay as calculated from the ED population was identical to that established by the manufacturer arguing for the robustness and usefulness of this assay in the ED.

It has been reported that elderly more often present with unspecific complaints[26]. Our study confirms these findings since the relative number of patients presenting with isolated dyspnea or other unspecific complaints increased considerably in the elderly. In turn, this observation supports the earlier recommendations to consider NSTEMI in patients with unspecific complaints when they are > 75 years old, although this is not anymore specifically outlined in the latest ESC guideline[3] but was well described in former ones[26].

## Limitations

Patients discharged from the hospital were not followed-up. Thus, it cannot be excluded that patients were discharged but were re-hospitalized later due to ACS. Furthermore, subpopulations stratified for eGFR differed in size. The patient group with eGFR < 30 mL/min/1.73m^2^ comprised only 184 individuals compared to 1376 with normal renal function. The proportion of patients with final diagnosis of MI is 6.1% which is somewhat lower compared to other reports [17,20,27,28]. This may limit the power to compare the diagnostic performance of POC versus hs-cTn assays. Likewise, the present population may be suboptimal to draw conclusions with respect to the impact of renal dysfunction on the diagnosis of MI. However, it is clear from the present results that elevated cTn values are associated with reduced kidney function which in turn may have crucial impact on the outcome of the initial assessment of patients admitted to the ED. According to the Third Definition of MI, the serial measurement of cTn is inevitable for the delta detection which is the basis for rule-in or rule-out of MI. The present study, however, investigated only one time-point, i.e. at admission, without taking into account the delta detection or time elapsed from onset of chest to admission to the ED. To overcome this limitation, the study uses the release diagnosis as a criterion for the assessment of the diagnostic performance of different cTn assays at admission of patients to the ED in a retrospective manner.

## Conclusion

All three cTn assays demonstrated equivalent diagnostic performance based on ROC analysis in non-selected ED-patients using the initial cTn value at admission to the ED in relation to the release diagnosis. This supports the use of cTn POC testing in this setting. Because cTnT concentrations from both core laboratory as well as POC were associated with decreased renal function as assessed by eGFR, our data implicate that the use of additional cut-off values depending on renal function may improve patient care. Thereby, the diagnostic performance would be increased and the number of false positive measurements may be reduced avoiding expensive, unnecessary treatments.

## Supporting information

S1 Table Frequencies of test outcomes using the optimized cut-off (OCO) value as determined by receiver operating characteristic (ROC) analyses. (DOCX)

S2 Table Frequencies of test outcomes using the manufacturers recommended cut-off values (CO mf). (DOCX)
