# Supplementary material for: Diagnostic performance of point-of-care and central laboratory cardiac troponin assays in an emergency department
Source: PLoS One. 2017 Nov 28;12(11):e0188706. doi: 10.1371/journal.pone.0188706 (PMC5705122; doi:10.1371/journal.pone.0188706)
Supplement: S1 Table — (DOCX) [file pone.0188706.s001.docx]

**S1 Table: Frequencies of test outcomes using the optimized cut-off (OCO) value as determined by receiver operating characteristic (ROC) analyses.**

|  | **OCO** | **TP** | **TN** | **FP** | **FN** | **PPV (%)** | **NPV (%)** |
| --- | --- | --- | --- | --- | --- | --- | --- |
| **eGFR ≥ 60** | **n = 75/1376** | | | | | | |
| Lab hsTnT (ng/L) | 33 (18; 36) | 58 | 1218 | 83 | 17 | 41 (19; 50) | 99 (98; 99) |
| POC TnT (ng/L) | 18 (13; 22) | 60 | 1152 | 149 | 15 | 29 (19; 37) | 99 (98; 99) |
| POC TnI (ng/L) | 14 (14; 27) | 62 | 1175 | 126 | 13 | 33 (27; 55) | 99 (98; 99) |
| **30 ≤ eGFR < 60** | **n = 38/603** | | | | | | |
| Lab hsTnT (ng/L) | 33 (29; 43) | 32 | 434 | 131 | 6 | 20 (14; 31) | 99 (97; 100) |
| POC TnT (ng/L) | 33 (21; 38) | 27 | 483 | 82 | 11 | 25 (12; 34) | 98 (97; 99) |
| POC TnI (ng/L) | 15 (11; 24) | 27 | 453 | 112 | 11 | 19 (12; 34) | 98 (96; 99) |
| **eGFR < 30** | **n = 12/184** | | | | | | |
| Lab hsTnT (ng/L) | 100 (100; 202) | 11 | 142 | 30 | 1 | 27 (15; 47) | 99 (98; 100) |
| POC TnT (ng/L) | 97 (97; 190) | 11 | 145 | 27 | 1 | 29 (15; 46) | 99 (98; 100) |
| POC TnI (ng/L) | 46 (13; 180) | 9 | 150 | 22 | 3 | 29 (4; 48) | 98 (94; 100) |

Patients were stratified in three groups according their renal function. For each patient group the number of people diagnosed with myocardial infarction in relation to the number of overall patients is given. The optimized cut-off value (OCO) was determined by ROC analyses. The number of true positives (TP), true negatives (TN), false positives (FP), and false negative (FN) is given. Additionally the positive predicted value (PPV) as well as negative predicted value (NPV) is presented. In brackets the 95% confidence interval for each parameter is given. Confidence intervals were assessed by using bootstrapping methods. eGFR = estimated glomerular filtration.
